# A new prediction diagnosis model of incomplete Kawasaki disease based on data mining with big data

**DOI:** 10.1002/pdi3.2516

**Published:** 2024-12-23

**Authors:** Zhen Yang, Bo Pan, Jia Liu, Haolin Wang, Jie Tian

**Affiliations:** ^1^ Department of Cardiology Children's Hospital of Chongqing Medical University Chongqing China; ^2^ Key Laboratory of Children's Vital Organ Development and Diseases of Chongqing Health Commission Chongqing China; ^3^ National Clinical Key Cardiovascular Specialty Chongqing China; ^4^ College of Medical Informatics Chongqing Medical University Chongqing China

**Keywords:** age grouping, data mining, incomplete Kawasaki disease, independent risk factors, prediction model

## Abstract

Kawasaki disease (KD) is an acute, self‐limited febrile illness occurring in children. In actual clinical situations, unlike complete Kawasaki disease (CKD), incomplete Kawasaki disease (IKD) lacks typical symptoms and is difficult to distinguish from many febrile illnesses, which poses a challenge to accurate diagnosis and misleading the treatment. Therefore, we investigated the independent risk factors for early prediction of IKD in children. In this research, 809 children suffering from IKD were recruited from the Children's Hospital of Chongqing Medical University from 2007 to 2017, as well as 2427 children were related to febrile diseases, divided into the IKD group and the other related febrile disease group. According to the results of univariate analysis, the study population was divided into three age groups to develop group‐specific models that demonstrated more effective performance. Finally, the 0–24 months old group obtained eight independent risk factors: CRP, LDH, UA, TP, ALB, RDA, PLT, and HGB, with the ROC curve showing an AUC of 0.862 in the predictive model and 0.88 in the new dataset. Meanwhile, LDH, UA, ALB, PLT, and MCHC were in the 24–60 months old group, among which AUC was 0.83 in the predictive model and 0.82 in the new dataset; the older group obtained LDH, UA, MCHC, and PLT, with an AUC of 0.7 in the predictive model and 0.8 in the new dataset. Particularly, UA is a new independent risk factor of IKD. These findings offer valuable insights into guiding the personalized diagnosis of IKD in pediatric patients.

## Graphical abstract

In this study, based on a medical big data information mining approach, we mined the independent risk factors for incomplete Kawasaki disease prediction by comparing incomplete Kawasaki disease with related febrile illnesses and constructed an auxiliary diagnostic model used to predict incomplete Kawasaki disease.

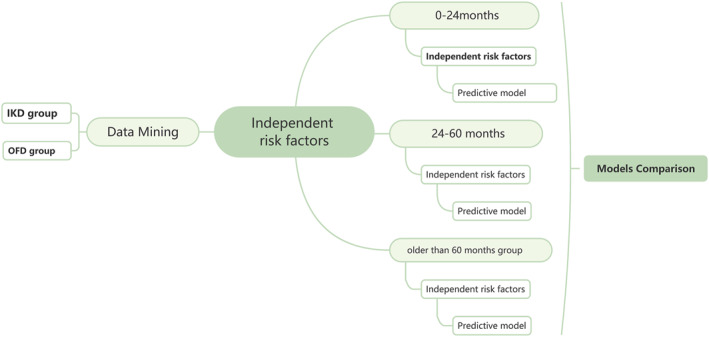

## 1 INTRODUCTION

Kawasaki disease (KD) is an acute self‐limited febrile disease that occurs in children under 5 years old, and it has been the second most important cause of acquired heart disease in children after acute rheumatic fever (ARF).^1^ However, the etiology of KD is still currently unclear. Most researchers consider that the occurrence of KD was influenced by both hereditary and environmental factors.^2^ Epidemiological investigation indicates variations in susceptibility of KD among different ethnicities. The incidences of KD were high in Southeast Asia, and Japan was the area with highest incidences, while the rate of KD in European countries and America was relatively low.^3^ In recent years, the incidence of KD has increased, and it has become urgent to investigate the pathogenesis and risk factors.

KD, also known as mucocutaneous lymph node syndrome, was divided into complete Kawasaki disease (CKD) and incomplete Kawasaki disease (IKD). The diagnosis of CKD depends on clinical symptoms, including fever, conjunctival hyperemia, oral changes (strawberry tongue), rash, cervical lymphadenopathy, erythema of the palms and soles, and firmness.^4^ However, these typical symptoms are not commonly observed in clinical practice among children with IKD, which increases the difficulty in diagnosis.^3^, ^5^ It might be difficult to identify and diagnose IKD at an early stage with its unusual clinical symptoms and restricted expression in children, leading to the occurrence of serious coronary artery complications ultimately.

A previous study shows that the prognosis of IKD is more challenging than that of CKD.^6^ In the clinical comparison with children with KD, the coronary artery damage rate in the IKD group was strikingly higher than that in the CKD group, and the incidences of abnormal ECG and coronary artery disease were also alarmingly higher in the IKD group than those of the CKD group. However, compared with children of the CKD group, children of the IKD group had lower clinical symptoms and were more likely to be misdiagnosed.^7^, ^8^ An enormous amount of clinical research studies have been conducted to increase the KD diagnostic accuracy in the past decades. Nevertheless, there is no separate diagnostic model for IKD.

Given the current state of the literature and the observation that children with IKD develop the disease at a younger age and have a more severe course of illness in newborns and young children, it is not possible to accurately represent this characteristic using a single model created from the entire dataset. Thus, the study population was divided into three age groups to create age‐specific models that demonstrated superior performance, as judged by our analysis. In this study, we collected and retrospectively analyzed the cases of IKD and other related febrile diseases (RFD) in the Children's Hospital of Chongqing Medical University during the past decades, and we constructed a prediction model for the diagnosis of IKD.

## 2 METHODS

### 2.1 Data collection

This study selected 810 inpatients from the Children's Hospital of Chongqing Medical University who were diagnosed with IKD and discharged from the hospital between October 2007 and December 2017. The control group selected 4725 cases of other related febrile diseases among hospitalized children in the same hospital from 2016 to 2018. These febrile diseases are easily confused in the diagnosis of admission and discharge. Among them, 50 cases will be selected every month if the monthly cases of each disease are more than 50 cases, and disease cases that are less than 50 cases will be selected. Other febrile diseases are acute tonsillitis, upper respiratory tract infection, bronchitis, bronchial pneumonia, adenovirus infection, pharyngeal conjunctival fever, lymphadenitis, hand, foot, and mouth disease, measles, infectious mononucleosis, acute rash in children, scarlet fever, urinary tract infection, and sepsis.

The data contain all the information during the hospitalization of the child and are encrypted. The unique identification number of all children is the child ID. All extracted information was imported into SQL2008 in an Excel form for cleaning.

Information collected includes 190 items of epidemiological data, laboratory data, and imaging data of all children in the IKD group, and other febrile disease groups were collected. 1) Epidemiological data included the following: gender, month age, hospitalization days, and hospitalization expenses. 2) Laboratory test data included the following: blood routine, CRP, liver function, kidney function, electrolytes, erythrocyte sedimentation rate, myocardial enzyme spectrum, stool routine, urine routine, procalcitonin, antigen antibodies of various pathogenic microorganisms, autoimmune antibodies, etc. and each item under each inspection.

We have collected the case data of children with IKD (607 cases) and RFD group diseases (1821 cases) newly admitted from 2018 to 2020 in the same hospital for model validation. All extracted information was imported into SQL2008 for cleaning.

### 2.2 Model establishment

Randomly select 80% of the dataset to construct a predictive model, and use the remaining 20% of the dataset as a test set. Use OR value, *p*‐value, lasso analysis, and other methods to screen independent risk factors, and build a multivariate logistic regression model. The model is independent, protective, and optimized by age subgroups, and the independent risk factors of each age group are screened again, and the new multivariate logistic regression equation was constructed.

The study uses Python for data analysis. The count data are expressed as a percentage, and the measurement data are expressed as the mean ± standard deviation. If *p* < 0.05, it means that there is a significant statistical difference between the two groups. Variables with significant statistical differences were included in the lasso and multivariate logistic regression analysis, and the OR value and 95% confidence interval of each variable were calculated at the same time. The independent risk factors for IKD were finally included in the regression equation. The receiver operating curve (ROC) and area under the curve (AUC) were used to calculate the authenticity predictive power of the model. Using sensitivity and specificity to reflect the predictive power of the model.

The study population was divided into three age groups: 0–24 months old group, 24–60 months old group, and older than 60 months old group. In the three models, a positive variable coefficient is a risk factor for IKD, and a negative variable coefficient is a protective factor for IKD. The probability of occurrence of IKD is calculated by the regression equations of each group. If the probability of IKD > 0.05, it means that the child could have probably suffered from IKD.

## 3 RESULTS

### 3.1 Univariate analysis of whole age

After data cleaning, the two groups of variables were analyzed initially. The results of single‐factor analysis retained 44 indicators with *p* < 0.05 (the result is shown in Appendix, Table A1, including blood routine, liver and kidney function, urine routine, stool routine and CRP), absolute value of red blood cell distribution (RDA), red blood cell distribution width (RDW), phosphorus, sodium ion, average hemoglobin amount, average platelet volume, absolute lymphocyte value, uric acid (UA), globulin (GLB), ketone body, absolute value of neutrophils, albumin (ALB), large platelet ratio, absolute value of monocytes, aspartate aminotransferase (AST), red blood cells, creatinine, potassium, alkaline phosphatase (ALP), percentage of lymphocytes, magnesium, hemoglobin (HGB), platelet distribution width, hematocrit, total protein (TP), total calcium, white blood cells (WBC), glutamyl transpeptidase (GGT), aspartate, hematocrit, chloride ion (Cl), urea nitrogen (BUN), average red blood cell volume, lactate dehydrogenase (LDH), platelet count (PLT), percentage of neutrophils, total bilirubin (TBIL), white blood cell morphology, specific gravity, stool characteristics, urine glucose, and month age.

### 3.2 Independent risk factors and model prediction of whole age

The variables with significant statistical differences in the univariate analysis were included in the lasso analysis, and 14 independent risk factors were obtained: CRP, RDA, UA, GLB, ALB, AST, ALP, HGB, GGT, Cl, LDH, PLT, TBIL, and age of the month. According to the selected independent risk factors, the logistic regression model is obtained.

Log−oddsofIKD=14.953+0.002×CRP−0.103×RDA−0.004×UA+0.028×GLB−0.077×ALB+0.000×AST−0.001×ALP−0.025×HGB+0.003×GGT−0.052×Cl−0.004×LDH+0.002×PLT−0.012×TBIL−0.015×AGEOFMONTH

**FIGURE A1:**
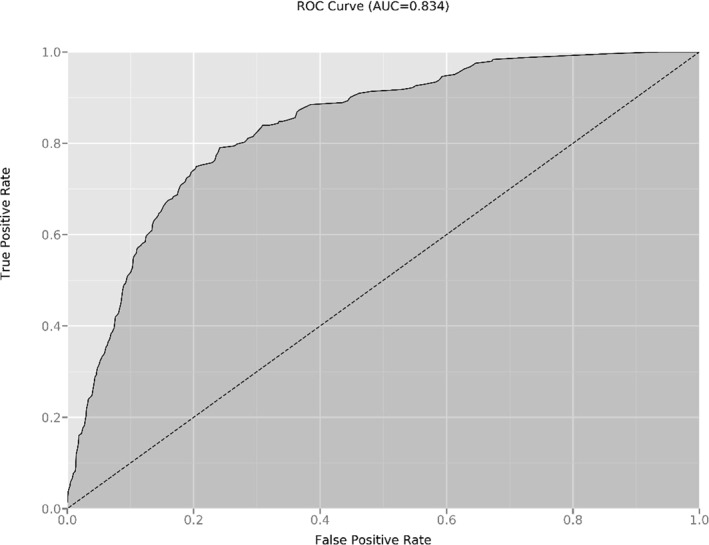
ROC of the prediction model.

The ROC and AUC are used to calculate the predictive performance of the model. The ROC curve of the prediction model is shown in Appendix, Figure A1. The AUC of the prediction model test set is 0.834, and the 95% AUC confidence interval obtained by the bootstrap resampling method is 0.805–0.854.

**FIGURE A2:**
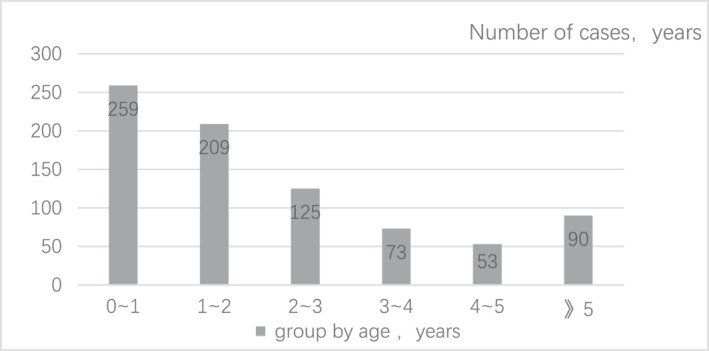
Age distribution from model building data.

Through the risk factor age of month we had obtained, we reorganized the data and found that the majority of IKD patients in the database were under 2 years old, while there were fewer patients over 5 years old (Appendix, Figure A2). Since the AUC value of the constructed prediction model is less than 0.85, the performance of the best prediction model has not been reached. Therefore, the model was rebuilt based on the above analysis and data results by age. In addition, in the first part of the analysis results, there is a mismatch in the amount of data between the two groups, which leads to the phenomenon of deviation of the model. To eliminate the effects of age and solve the class imbalance problem, a matched subset of the RFD group was examined with propensity scoring matching for the IKD cohort in each age group. The ratio number of matched samples of IKD to RFD group is 1:3.

### 3.3 Univariate analysis of each age group

After the IKD group and the RFD group were divided by age, different univariate analysis results were obtained (Appendix, Table A2).

There are 44 variables in the 0–24 months age group, 40 variables in the middle group, and 31 variables in the older group, which were all different from the result of the first part.

### 3.4 Independent risk factors of each age group

In the univariate analysis results of the two groups of variables in the three age groups, the variables with significant statistical differences were included in the lasso analysis. The selected variables with *p* < 0.05 were brought into logistic regression analysis again, and the independent risk factors of the three age groups were obtained as shown in Tables 1, 2, 3.

**TABLE 1:** The OR values of the independent risk factors for IKD: 0–24 months old group.

Indicators after lasso	IKD (*N* = 468)	RFD (*N* = 1404)	The first logistic regression analysis			Second logistic regression analysis		
			OR	95% confidence interval	*p*	OR	95% confidence interval	*p*
CRP, mg/L	28.0 (10.0–51.0)	4.0 (4.0–12.0)	1.010	(1.005,1.015)	<0.001	1.010	(i.006,1.015)	<0.001
LDH, U/L	284.6 (242.5–339.0)	343.3 (288.0–437.0)	0.997	(0.996,0.998)	<0.001	0.997	(0.995,0.998)	<0.001
UA, umol/L	192.0 (150.0–246.0)	240.0 (187.0–304.0)	0.995	(0.994,0.997)	<0.001	0.995	(0.994,0.997)	<0.001
TP, g/L	59.6 (54.5–64.2)	61.4 (57.3–65.4)	1.069	(1.038,1.101)	<0.001	1.071	(1.040,1.102)	<0.001
ALB, g/L	38.2 (35.0–41.2)	42.9 (40.1–45.5)	0.856	(0.821,0.893)	<0.001	0.852	(0.817,0.888)	<0.001
RDA, fL	40.0 (38.0–43.0)	40.0 (38.0–43.0)	0.949	(0.916,0.983)	0.004	0.937	(0.907,0.967)	<0.001
PLT, *10^9^/L	455.0 (347.0–607.0)	311.5 (213.0–423.0)	1.003	(1.002,1.004)	<0.001	1.003	(1.002,1.004)	<0.001
HGB, g/L	100.0 (93.0–109.0)	112.0 (103.0–118.0)	0.973	(0.959,0.987)	<0.001	0.977	(0.964,0.990)	<0.001
MCHC, g/L	324.0 (317.0–332.0)	326.0 (317.0–333.0)	1.009	(0.997, 1.022)	0.143	/	/	/
TBIL, umol/L	5.6 (3.6–7.8)	5.5 (3.8–8.1)	0.992	(0.976, 1.008)	0.323	/	/	/

**TABLE 2:** The OR values of the independent risk factors for IKD: 24–60 months old group.

Indicators after lasso	IKD (*N* = 251)	RFD (*N* = 753)	The first logistic regression analysis			Second logistic regression analysis		
			OR	95% confidence interval	*p*	OR	95% confidence interval	*p*
LDH, U/L	263.0 (229.8–314.3)	294.6 (250.1–377.5)	0.997	(0.996,0.999)	0.001	0.997	(0.995,0.999)	<0.001
UA, umol/L	195.0 (159.0–243.4)	238.0 (195.0–307.0)	0.993	(0.991,0.996)	<0.001	0.993	(0.990,0.996)	<0.001
ALB, g/L	39.2 (35.5–41.2)	42.7 (39.6–45.5)	0.866	(0.817,0.917)	<0.001	0.831	(0.796,0.8670	<0.001
PLT, *10^9^/L	366.0 (278.0–458.0)	266.0 (205.0–341.0)	1.004	(1.003,1.006)	<0.001	1.004	(1.003,1.006)	<0.001
MCHC, g/L	329.0 (323.0–336.0)	328.0 (321.0–336.0)	1.042	(1.024,1.060)	<0.001	1.039	(1.023,1.056)	<0.001
CRP, mg/L	25.0 (4.0–62.0)	4.0 (4.0–18.0)	1.004	(0.999, 1.010)	0.117	/	/	/
TP, g/L	61.5 (58.0–65.5)	64.8 (61.1–68.5)	0.981	(0.942, 1.021)	0.352	/	/	/
HGB, g/L	108.0 (100.0–115.0)	116.0 (109.0–123.0)	0.988	(0.968, 1.009)	0.253	/	/	/

**TABLE 3:** The OR values of the independent risk factors for IKD: >60 months old group.

Indicators after lasso	IKD (*N* = 90)	RFD (*N* = 270)	OR	95% confidence interval	*p*
LDH, U/L	228.0 (199.0–300.3)	290.0 (228.0–405.6)	0.996	(0.993, 0.998)	0.002
UA, umol/L	195.0 (164.0–249.0)	245.0 (202.0–308.3)	0.992	(0.988, 0.997)	0.001
PLT, *10^9^/L	324.0 (247.0–448.0)	257.0 (194.0–356.0)	1.003	(1.001, 1.006)	0.009
MCHC, g/L	331.0 (323.0–336.0)	329.0 (320.0–335.0)	1.041	(1.006, 1.077)	0.022
CRP, mg/L	25.0 (12.0–62.0)	14.0 (4.0–51.0)	0.999	(0.992, 1.006)	0.743
TP, g/L	63.8 (59.8–69.0)	65.8 (61.7–69.9)	0.998	(0.948, 1.052)	0.948
ALB, g/L	39.0 (35.2–41.0)	40.7 (36.6–43.6)	0.946	(0.872, 1.026)	0.183
RDA, fL	39.0 (38.0–41.0)	40.0 (38.0–43.0)	0.941	(0.857, 1.034)	0.205
HGB, g/L	111.0 (103.0–118.0)	117.0 (106.0–126.0)	0.981	(0.952, 1.011)	0.217

In the 0–24 months age group, there are eight independent risk factors, and five independent risk factors in the middle group. In the analysis of the group older than 60 months, it was found that there were fewer IKD case data, and fewer independent risk factors were screened out by regression analysis.

### 3.5 Predictive model construction of each age group

According to the independent risk factors of the three age groups, the predictive models were constructed respectively.

A total of eight independent risk factors including CRP, LDH, UA, TP, ALB, RDA, PLT, and HGB, were screened in the 0–24 months age group for the construction of the logistic regression equation. The regression equation for the 0–24 months age group was obtained as follows:

Log−oddsofIKD1=7.25+0.010×CRP−0.003×LDH−0.05×UA+0.068×TP−0.160×ALB−0.065×RDW+0.003×PLT−0.023×HGB

A total of 5 independent risk factors including LDH, UA, ALB, PLT, and MCHC, were screened out in the 24–60 months old group for the construction of the logistic regression equation, and the regression equation for the 24–60 months old group was obtained as follows:

Log−oddsofIKD2=0.038×MCHC−0.003×LDH−0.007×UA−0.186×ALB+0.004×PLT−5.139

There are fewer independent risk factors in the group older than 60 months. All the 9 indicators screened by lasso are included in the equation for calculation, and the following equation is obtained:

Log−oddsofIKD3=0.040×MCHC+0.003×PLT−0.001×CRP−0.005×LDH−0.008×UA−0.002×TP−0.055×ALB−0.060×RDW−0.019×HGB−5.309

Through the above three sets of formulas, the probability of IKD in children can be calculated.

### 3.6 Predictive model validation of each age group

To evaluate our model's predictive performance and practical utility comprehensively, we use calibration curve, decision curve, and ROC curve for analysis.

The analysis results of the calibration curve and the decision curve indicate that our grouping model has good predictive performance and practicality utility (Appendix Table A3).

According to the selected independent risk factors and the prediction model, the newly admitted children were been predicted in the same period. Substituting each independent risk factor into the prediction model for simultaneous verification, and comparing with the test set data in the original data, as shown in Table 4.

**TABLE 4:** Validation results of the three models incorporate new data.

	0–24 months		24–60 months		>60 months	
	20% test set	New data (*N* = 337:1011)	20% test set	New data (*N* = 183:549)	20% test set	New data (*N* = 87:262)
Test AUC	0.862	0.878	0.829	0.818	0.694	0.804
Youden	0.613	0.623	0.535	0.51	0.333	0.494
Sensitivity	0.851	0.757	0.840	0.749	0.722	0.897
Specificity	0.762	0.866	0.695	0.761	0.611	0.598
Precision	0.544	0.654	0.477	0.511	0.382	0.426
Accuracy	0.784	0.839	0.731	0.758	0.639	0.672

The AUC value of the prediction model in the 0–24 months age group is 0.862 in the test set and 0.878 in the new data verification result; its sensitivity and specificity are 0.851 and 0.762 in the test set, and 0.757 and 0.866 in the new data verification, respectively.

The AUC value of the prediction model in the 24‐60‐month‐old group is 0.829 in the test set and 0.818 in the new data verification result; its sensitivity and specificity are 0.840 and 0.695 in the test set, and 0.749 and 0.761 in the new data verification, respectively.

The AUC value of the prediction model in the 60‐month‐old group was 0.694 in the test set and 0.804 in the new data verification result; its sensitivity and specificity were 0.722 and 0.611 in the test set, and 0.897 and 0.598 in the new data verification, respectively.

### 3.7 Models’ comparison

In the currently published literature, there is no prediction model specifically for IKD, so this model will be compared with the published KD prediction model in the same data.^9^, ^10^, ^11^ The model comparison results are shown in Table 5; some of them without sufficient useful data are not included. (Explanation: Since the amount of data in the 60‐month‐old group was relatively small, the training set and the testing set were re‐divided for debugging and verification when comparing other models. Consequently, there are slight differences between the data results and the previous ones [Table 4]).

**TABLE 5:** Comparison parameters of similar prediction models.

	0–24 months			24–60 months			>60 months		
	AUC	Sensitivity	Specificity	AUC	Sensitivity	Specificity	AUC	Sensitivity	Specificity
Our model	0.862	0.851	0.762	0.829	0.840	0.695	0.841	0.833	0.778
Huang model	0.845	0.819	0.740	0.795	0.700	0.795	0.753	0.889	0.556
Liu model	0.778	0.670	0.797	0.760	0.740	0.689	0.631	0.611	0.741
Falcini model	0.770	0.660	0.794	0.658	0.580	0.828	0.692	0.778	0.611
Barone model	0.769	0.649	0.811	0.737	0.600	0.828	0.699	0.500	0.852
Ling model	0.750	0.670	0.762	0.675	0.520	0.874	0.688	0.722	0.667

**FIGURE 1:**
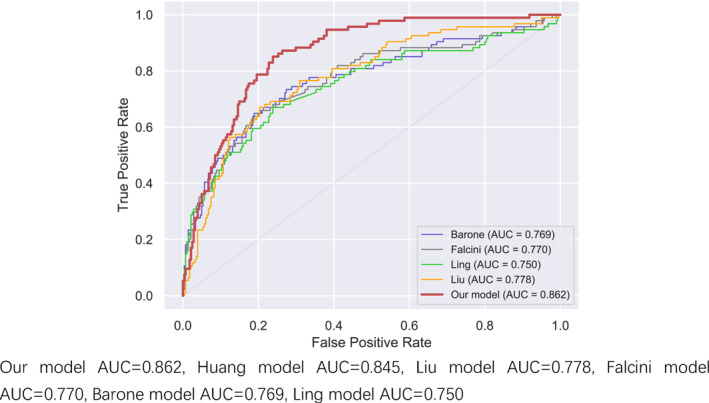
0–24 months ROC curve.

**FIGURE 2:**
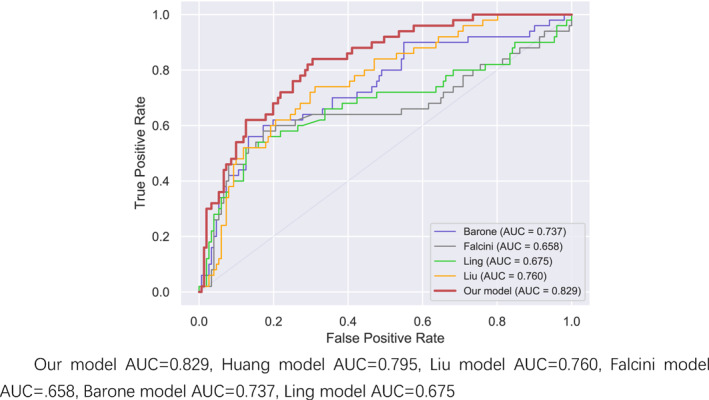
24–60 months ROC curve.

**FIGURE 3:**
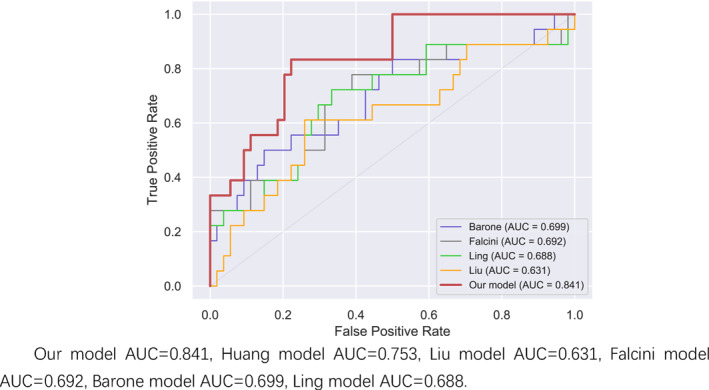
Older than 60 months ROC curve.

In the comparison of AUC results in Figures 1, 2, 3 in the 0–24 months age group, the AUC of our model is 0.862, which is higher than the Huang prediction model with AUC of 0.845 in the same region.^9^ (Explanation: This model aims to focus on the diagnosis of IKD in children from different regions. Since the yellow model belongs to the same region as our model, only its data is displayed instead of being depicted in the curve graph.) In terms of sensitivity and specificity, the comprehensive comparison of the three‐stage models in this study is higher than other models, especially in the 0–24‐month‐old group.

In the age group older than 60 months, due to the small amount of data, the nine indicators screened out by lasso are included for modeling analysis. Compared with similar models, the AUC obtained by it is higher than that of other models, and the result of sensitivity and specificity is also higher than other models.^12^, ^13^, ^14^

It can also be seen in the ROC curve that the models of the three age groups are more stable than other similar models, especially in the younger age group, as shown in Table 5.

### 3.8 Model nomogram

**FIGURE 4:**
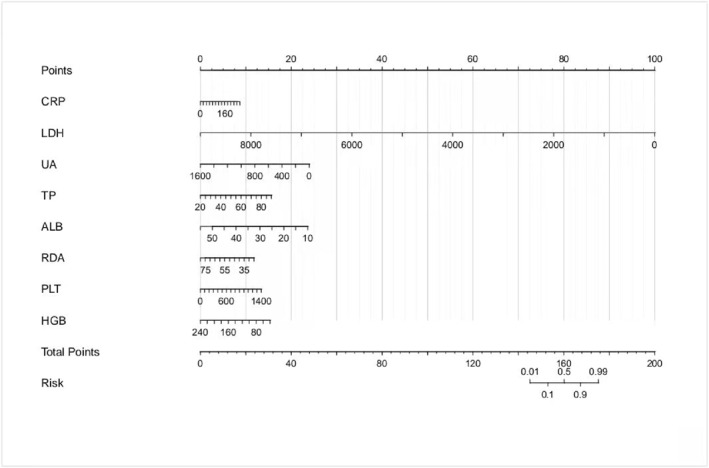
0–24 months model nomogram.

**FIGURE 5:**
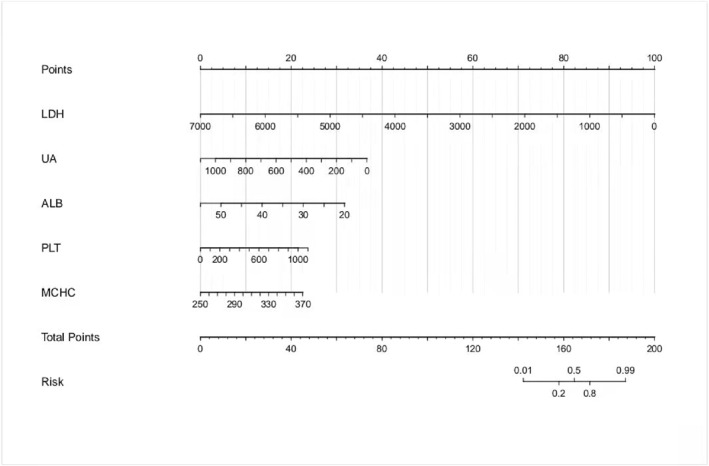
24–60 months model nomogram.

**FIGURE 6:**
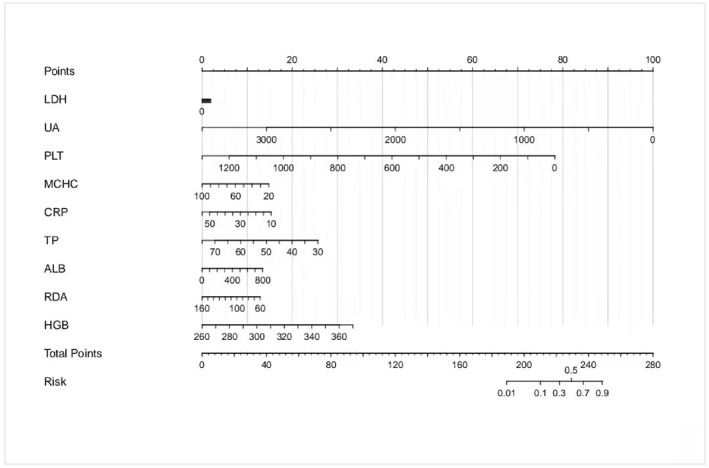
>60 months model nomogram.

Nomogram as an easy‐to‐understand tool for clinical application can aid doctors in using our model effectively in clinical settings. Thus, we design nomograms for our models based on logistic regression equations of each group as shown in Figure 4, 5, 6.

## 4 DISCUSSION

### 4.1 Independent risk factors

There are 44 independent risk factors obtained in the first part, which are very similar to the 0–24‐month‐old group, but the AUC values obtained from the model are significantly different. It is known that the reason is related to the data of the larger group of children through the construction of the age group model. The final independent risk factors were different for the three age groups.

The relative protective factors for the three age groups are LDH and UA. Among them, LDH has been found in previous KD studies.^15^ The newly discovered independent risk factor is UA, an independent risk factor that exists in the three age groups, the OR < 1, and the *p* < 0.05, which is a protective factor relative to the OFD group. In a study of UA in an area of China,^16^ it was found that compared with upper respiratory tract infection, hand, foot, and mouth disease, and nephritis, the UA level of children with KD was relatively lower, and the difference was statistically significant, which was consistent with the results of our study. There are many studies on the effects of gout caused by hyperuricemia and cardiovascular disease in adults,^17^ but the study on UA in children is still unclear. Some studies have shown child obesity and diabetes may be associated with UA, but in the KD of systemic vasculitis, the level of UA in the same age group is lower than that of other febrile diseases, which may be related to the antioxidant effect of UA.^18^, ^19^

The relative risk factor for the three age groups was PLT, which was consistent with the KD diagnosis and treatment guidelines.

In the group less than 24 months of age, independent risk factors with OR < 1 include ALB, RDA, and HGB in addition to LDH and UA. Among them, ALB and HGB are related to the occurrence of IVIG resistance, and HGB has also reported that their decline may be related to the occurrence of IKD.^20^, ^21^, ^22^, ^23^ Independent risk factors with OR > 1, include CRP, TP, and PLT. Among them, CRP and PLT have been clearly mentioned in the KD guidelines and confirmed in reports. They are used clinically to diagnose KD and determine the prognosis of the disease.^23^, ^24^

In the 24–60‐month‐old group and the 60‐month‐old group, the independent risk factors with OR > 1 and *p* < 0.05 were PLT and MCHC. MCHC did not appear as an independent risk factor in the first part of the analysis of whole age groups. However, in the 24–60‐month‐old group and the 60‐month‐old group, the OR value of MCHC was greater than 1, and *p* < 0.05, which was different from the previous analysis of the first part of the whole age group.

MCHC is related to HGB but has different characteristics. According to research, HGB changes in children around 1‐year old are not obvious, but it changes with age. The difference is that MCHC is relatively stable in children over 1‐year old.^25^

In a comparative analysis of the IKD group and the RFD group, in the 24–60‐month‐old group and the 60‐month‐old group, MCHC existed as an independent risk factor, while HGB was not screened as an independent risk factor. It may also be related to the occurrence of disease, and its overall trend is in line with the law of growth and development of children.

### 4.2 Models

The models of the three age groups performed well in the test set and new data validation. The AUC has reached 0.8 or more in the new data verification, especially in the small children aged 0–24 months; the AUC were 0.862 and 0.878, both greater than 0.85, indicating that the predictive model is very effective. There are also intuitive responses in the ROC curve, which are more stable. Compared with previous KD prediction models, the three models in our study have obvious advantages in terms of sensitivity and specificity. Comparing the Huang model in the same region, the AUC of the Huang model is less than 0.8 in both the 24–60 month‐old group and the 60‐month‐old group. From the perspective of sensitivity and specificity, the prediction effect of the new model for the three age groups is more stable. The prediction effect of other models is not very good, and it may also be related to regional differences and ethnic differences.

## 5 INNOVATION AND MEANING

Our research is the first one to build a model from age segmentation. To adapt to the changes in children's growth and development, a segmented model is adopted, which is better suitable for children with KD. At the same time, it has better applicability and accuracy for children younger than 24 months of age who are prone to IKD. The purpose of the research is to solve the diagnosis difficulties caused by many factors such as atypical symptoms and poor self‐reporting ability in young children. The research results are basically in line with expectations. In the younger age group model, the ROC curve, AUC, and specificity sensitivity after grouping have been greatly improved compared with the first part.

This study uses the database method to clean and extract data, avoiding errors, omissions, and deviations that may occur in the previous manual direct data processing and is more efficient and accurate. Through our medical big data platform, all the examinations of the children are included in the analysis, and information mining is carried out to obtain independent risk factors and predictive models. On the premise of not increasing the burden of examinations for children, a new auxiliary diagnosis method is provided for clinicians to improve the diagnosis rate of children with IKD newly diagnosed.

In the new data validation of the model, the three age models in this study have been well confirmed, but no prospective study has been conducted to further validate the model. The next step will be to verify and improve the model in multicenter collaboration.

## 6 CONCLUSION

1. Our study found 14 independent risk factors for IKD: CRP, RDA, UA, GLB, ALB, AST, ALP, HGB, GGT, Cl, LDH, PLT, TBIL, and AGEOFMONTH. Especially, UA is a new independent risk factor of IKD. The independent risk factors changed after month age grouping, and it may related to the month age of the children.
2. Multivariate logistic regression models were constructed for three age groups respectively which demonstrate better performance than a single model validated by the split test set and an independent dataset. Compared with other similar KD prediction models, our models have the highest AUC and more advantageous sensitivity and specificity.

## AUTHOR CONTRIBUTIONS

All authors contributed to the study's conception and design. Material preparation, data collection, and analysis were performed by Zhen Yang, Jia Liu, and Bo Pan. The first draft of the manuscript was written by Zhen Yang and all authors commented on previous versions of the manuscript. All authors read and approved the final manuscript.

## CONFLICT OF INTEREST STATEMENT

The authors have no relevant financial or nonfinancial interests to disclose.

## ETHICS STATEMENT

This study was approved by the ethics committee of Children's Hospital of Chongqing Medical University (2020/No 160‐1) following the Declaration of Helsinki.

## Data Availability

The data that support the findings of this study are available on request from the corresponding author. The data are not publicly available due to privacy or ethical restrictions.

## Appendix

**TABLE A1:** Univariate analysis.

Indicators					
RBC	PCT	LYMPH%	BUN	Ca	Stool property
RDA	P‐LCR	LYMPH#	KET	K	WBC appearance
RDW	MCH	TP	UA	CL	Age of month
MCV	HGB	ALB	TBIL	*p*	
HCT	WBC	GLB	AST	Na	
PLT	MONO#	CRP	GGT	Mg	
MPV	NEUT#	Cr	AST/ALT	GLU	
PDW	NEUT%	LDH	ALP	SG	

**TABLE A2:** The results of univariate analysis of the three age groups in the IKD group and the OFD group by age (*p* < 0.05).

0–24 months		24–60 months		>60 months	
Age of month	*p*	Age of month	*p*	Age of month	PDW
AST	RBC	AST	RBC	AST	PCT
BUN	RDW	BUN	RDW	BUN	MPV
CRP	RDA	CRP	HCT	CRP	PLT
NEUT#	HCT	NEUT#	Cr	NEUT#	HGB
NEUT%	Cr	NEUT%	WBC	NEUT%	AST/ALT
LDH	WBC	LDH	PDW	LDH	Mg
MONO#	PDW	MONO#	PCT	MONO#	SG
P‐LCR	PCT	MONO%	MPV	P‐LCR	Hematuria
UA	MPV	P‐LCR	PLT	UA	
MCV	PLT	UA	HGB	MCV	
MCHC	HGB	PH	ALT	MCHC	
MCH	ALT	MCV	GGT	TP	
TBIL	GGT	MCHC	AST/ALT	Ca	
TP	AST/ALT	TP	Na	CL	
Ca	KET	Ca	Mg	LYMPH%	
CL	Na	CL	GLU	ALB	
LYMPH%	Mg	LYMPH%	SG	RBC	
LYMPH#	Stool property	GLB		RDW	
GLB	SG	ALB		RDA	
ALB	WBC appearance	D‐BIL		HCT	
ALP	VitC	ALP		WBC	

**TABLE A3:** The analysis result of the calibration curve and the decision curve of three age group models.

	Calibration curves	Decision curve
0–24 months		
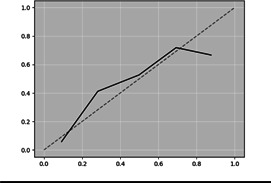
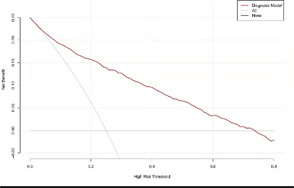
24–60 months		
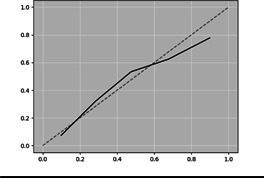
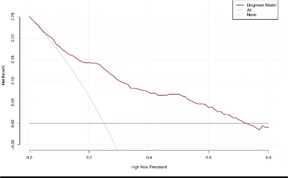
>60 months		
